# COVID-19 Computed tomography patterns in renal replacement therapy patients

**DOI:** 10.1590/2175-8239-JBN-2023-0029en

**Published:** 2024-03-11

**Authors:** Gabriel Assis Lopes do Carmo, Mariana Paiva Oliveira, Anna Luiza Lino Campos, Bráulio Roberto Gonçalves Marinho Couto, Lilian Pires de Freitas do Carmo, Tiago Lemos Cerqueira, Camila Alencar Monteiro de Souza, Yan Lopes Goll, Vitor Santos de Souza, Mariana Oliveira Guimarães Vieira, Pedro Alves Soares Vaz de Castro, Pedro Augusto Botelho Lemos, Ana Cristina Simões e Silva

**Affiliations:** 1 Hospital Evangélico de Belo Horizonte, Belo Horizonte, MG, Brazil.; 2 Universidade Federal de Minas Gerais, Faculdade de Medicina, Departamento de Clínica Médica, Belo Horizonte, MG, Brazil.; 3 Hospital Felicio Rocho, Belo Horizonte, MG, Brazil.; 4 Universidade Federal de Minas Gerais, Faculdade de Medicina, Belo Horizonte, MG, Brazil.; 5 Universidade Federal de Minas Gerais, Faculdade de Medicina, Laboratório Interdisciplinar de Investigação Médica, Unidade de Nefrologia Pediátrica, Belo Horizonte, MG, Brazil.

**Keywords:** Computed tomography, COVID-19, Dialysis, Emergency department, End stage kidney disease

## Abstract

**Introduction:**

Lung diseases are common in patients with end stage kidney disease (ESKD), making differential diagnosis with COVID-19 a challenge. This study describes pulmonary chest tomography (CT) findings in hospitalized ESKD patients on renal replacement therapy (RRT) with clinical suspicion of COVID-19.

**Methods:**

ESKD individuals referred to emergency department older than 18 years with clinical suspicion of COVID-19 were recruited. Epidemiological baseline clinical information was extracted from electronic health records. Pulmonary CT was classified as typical, indeterminate, atypical or negative. We then compared the CT findings of positive and negative COVID-19 patients.

**Results:**

We recruited 109 patients (62.3% COVID-19-positive) between March and December 2020, mean age 60 ± 12.5 years, 43% female. The most common etiology of ESKD was diabetes. Median time on dialysis was 36 months, interquartile range = 12–84. The most common pulmonary lesion on CT was ground glass opacities. Typical CT pattern was more common in COVID-19 patients (40 (61%) vs 0 (0%) in non-COVID-19 patients, *p* < 0.001). Sensitivity was 60.61% (40/66) and specificity was 100% (40/40). Positive predictive value and negative predictive value were 100% and 62.3%, respectively. Atypical CT pattern was more frequent in COVID-19-negative patients (9 (14%) vs 24 (56%) in COVID-19-positive, *p* < 0.001), while the indeterminate pattern was similar in both groups (13 (20%) vs 6 (14%), p = 0.606), and negative pattern was more common in COVID-19-negative patients (4 (6%) vs 12 (28%), *p* = 0.002).

**Conclusions:**

In hospitalized ESKD patients on RRT, atypical chest CT pattern cannot adequately rule out the diagnosis of COVID-19.

## Introduction

Coronavirus Disease 2019 (COVID-19), caused by Severe Acute Respiratory Syndrome Coronavirus 2 (SARS-CoV-2), was first described in Wuhan, China, in December 2019^1^. To date, the virus has infected more than 800 million people worldwide causing over 6 million deaths^2^. Similar to SARS-CoV and Middle East Respiratory Syndrome (MERS), symptoms are mainly respiratory, and severe forms account for up to 20% of cases^3^. Older age, obesity, hypertension, diabetes, underlying chronic cardiac, pulmonary and kidney diseases are clinical conditions related to worse prognosis^4,5^.

Patients with end stage kidney disease (ESKD) on renal replacement therapy (RRT) are of special concern, since they share many of these comorbidities and are highly exposed. These patients frequently travel to and from hemodialysis facilities and need to congregate several times a week in a closed environment^6^. Preliminary reports have shown an increased risk of death for this population^7,8,9^. Moreover, the clinical presentation of ESKD patients with respiratory diseases in the emergency department (ED) is also challenging, once they can have several underlying pulmonary conditions^10–12^. Such diseases may have similar clinical, laboratorial, and radiological findings that usually help diagnose COVID-19^13^. Therefore, it is crucial to define the radiological findings that allow early diagnosis of COVID-19 in patients with ESRD in the ED, in order to properly treat and isolate them. In this context, computed tomography (CT) is of particular importance for this evaluation. CT can show a typical pulmonary pattern that raises suspicion of SARS-CoV-2 infection, even when RT-PCR is negative^13^. Besides, sequential CTs during the patient’s disease course can detect complications and predict prognosis^14,15^.

The aims of this study were to describe pulmonary CT findings in patients with ESKD on RRT referred to ED with clinical suspicion of COVID-19, compare imaging characteristics of COVID-19-positive cases, confirmed by RT-PCR tests, with negative COVID-19 cases, and verify whether these CT results in patients with ESKD and COVID-19 have good sensitivity and specificity to diagnose COVID-19 without specific molecular tests. This analysis is part of the “Prospective study of CO**VI**D-19 in **d**ialytic p**a**tients (VIDA)”, which is currently recruiting cases (ReBEC number RBR-63hzd3, available at http://www.ensaiosclinicos.gov.br/rg/RBR-63hzd3/).

## Methods

### Study Design

The VIDA study is a multicenter retrospective and prospective cohort of patients with ESKD on RRT aiming to evaluate the impact of COVID-19 in this population. Individuals of both genders and older than 18 years have been recruited in 4 dialysis clinics of Associação Evangélica Beneficente de Minas Gerais, in Belo Horizonte, Brazil, since March 2020.

### Inclusion Criteria

The assistant nephrologist evaluated patients included in the VIDA cohort with suspicion of COVID-19 during the dialysis session to decide whether referral to the emergency department (ED) was needed. In this sub-analysis, we selected patients with respiratory symptoms referred to the ED between March and December 2020. During this period of time, the most prevalent viral lineages in Brazil were the B.1.1.28 and B.1.1.33^16^. No sample size calculation was performed since this was a convenience sample.

### Ethical Issues

The study was conducted in accordance to the Declaration of Helsinki. The local review board approved our study and all patients signed a written informed consent (Institutional Review Board number 31017120.9.3001.5149). The study is registered under the ReBEC number RBR-63hzd3, and the complete VIDA study protocol is available at http://www.ensaiosclinicos.gov.br/rg/RBR-63hzd3/.

### Study Protocol

**Figure 1.**
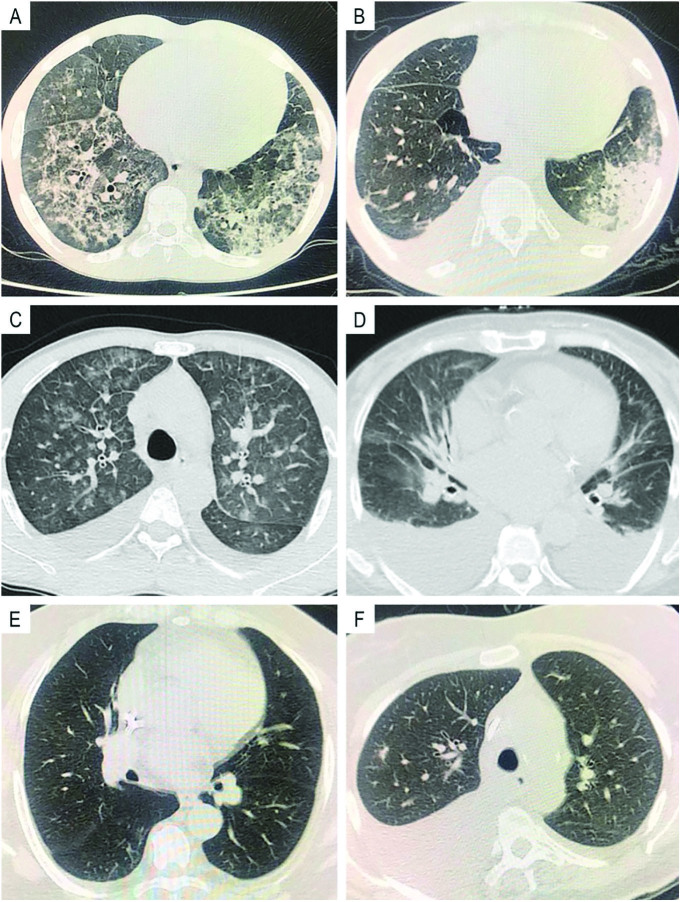
Computed chest tomography patterns in COVID-19. (A) Typical COVID-19: peripheral and bilateral ground glass opacity, consolidation and visible intralobular lines (“crazy-paving”); (B) Indeterminate: bilateral ground glass opacity especially in inferior lobes and pleural effusion; (C) Atypical: central ground glass opacity, bronchovascular bundle thickening and interlobular septal thickening, suggesting interstitial pulmonary edema; (D) Similar to image C plus pleural effusion and cardiomegaly, suggesting hydrostatic pulmonary edema; (E) Atypical: isolated consolidation with areas of ground glass opacities; (F) Negative: No CT features that suggest pneumonia.

Epidemiological baseline clinical information and vital signs were extracted from electronic health records. Based on these data, we calculated the Charlson score. We selected individuals who had a pulmonary CT scan and a nasopharyngeal swab for SARS-Cov-2 RT-PCR test to confirm the diagnosis of COVID-19. Pulmonary CT was interpreted by 2 experienced radiologists and classified as typical, indeterminate, atypical or negative for COVID-19 based on current guidelines (Figure 1)^17^. The primary analysis was the comparison of CT findings of positive and negative COVID-19 patients.

### Statistical Analysis

Study data were collected and managed using REDCap electronic data capture tools^18,19^. Numerical variables are presented as mean ± standard deviation (SD) or, in case of non-Gaussian distribution, as median values and ranges. Qualitative data are presented as percentage. Study groups (COVID-19+ versus COVID-19–) were compared in a univariate analysis by statistical tests of bilateral hypotheses, considering a 5% level of significance. We compared qualitative variables using Fisher’s exact or Chi-square tests. For quantitative data, Wilcoxon or Mann-Whitney tests were used. Sensitivity, specificity, positive predictive value (PPV), and negative predictive value (NPV), as well as ROC curves of the CT imaging patterns for confirmed COVID-19 diagnosis by RT-PCR were calculated. Variables with p-values ≤ 0.25 in the univariate analysis were included in a multivariate analysis by logistic regression to identify independent associations with a COVID-19 diagnosis. Associations were reported as odds ratio (OR) with their corresponding 95% confidence intervals (CI), as well as the test of statistical significance. We did not perform any statistical analysis for missing data. We used SPSS software (version 20) for all statistical analysis.

## Results

### Baseline Patient Characteristics

**Figure 2.**
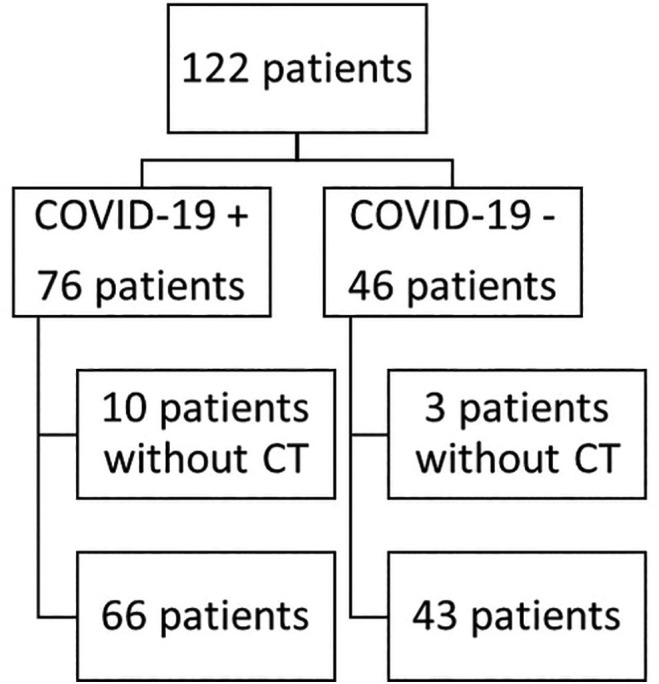
Enrollment flowchart.

From March to December 2020, 122 patients with ESKD on RRT were hospitalized at our institution with clinical suspicion of COVID-19. A total of 76 individuals (62.3%) tested positive for COVID-19, while the remaining 46 had another diagnosis (alternative group). Ten patients in the COVID-19 group and 3 in alternative group did not have chest CT and were excluded (Figure 2).

The clinical and demographic characteristics of the entire study cohort are summarized in Table 1. The mean age in the cohort was 60 ± 12.5 years, 43% were females, and the most common etiology of ESKD was diabetes, followed by hypertension. The median time on dialysis was 36 months (interquartile range (IQR) of 12-84), and the mean value of the prognostic Charlson score was 5 ± 34.1 (33% estimated 10-year survival). Clinical characteristics were similar in COVID-19-positive and COVID-19-negative patients, except for current tobacco use (0% vs 9%,*p* = 0.022, for COVID-19-positive and COVID-19-negative, respectively) (Table 1). The main alternative diagnosis for patients without COVID-19 was pulmonary congestion, followed by pneumonia and dialysis catheter-related bloodstream infection. Most patients had more than one diagnosis, as shown in Table 2.

**Table 1:** Baseline characteristics of the study population

Characteristic	All participants (109)	COVID-19 + (66)	COVID-19 – (43)	*p* value
**Age**	60 ± 12.5	60 ± 12.8	59 ± 12.3	0.794
**Female sex**	47 (43%)	27 (41%)	20 (47%)	0.693
**Hypertension**	103 (95%)	61 (92%)	42 (98%)	0.400
**NID Diabetes Mellitus**	10 (9%)	7 (11%)	3 (7%)	0.737
**ID Diabetes Mellitus**	50 (46%)	29 (44%)	21 (49%)	0.695
**Current Tobacco use**	4 (4%)	0 (0%)	4 (9%)	0.022
**Former tobacco use**	12 (11%)	8 (12%)	4 (9%)	0.761
**Coronary artery disease**	14 (13%)	6 (9%)	8 (19%)	0.158
**Heart failure**	16 (15%)	9 (14%)	7 (16%)	0.784
**Previous AMI**	9 (8%)	5 (8%)	4 (9%)	0.737
**Cerebrovascular disease**	15 (14%)	8 (12%)	7 (16%)	0.578
**Peripheral artery disease**	4 (4%)	2 (3%)	2 (5%)	0.646
**COPD**	8 (7%)	3 (5%)	5 (12%)	0.260
**Asthma**	5 (5%)	1 (2%)	4 (9%)	0.078
**AIDS**	1 (<1%)			
**Charlson Score**				
**Numerical**	5 ± 1.8	5 ± 1.8	5 ± 1.7	0.968
**Percentual**	32 ± 33.7	32 ± 33.6	32 ± 34.3	0.950
**Charlson Score (%)**	33 ± 34.1			
**Etiology of CKD**				
**Diabetes**	50 (41%)	32 (42%)	18 (39%)	0.145
**Hypertension**	27 (22%)	15 (20%)	12 (26%)	
**Glomerulopathy**	15 (12%)	13 (17%)	2 (4%)	
**Other**	30 (25%)	16 (21%)	14 (30%)
**Time on dialysis (months)**	36 [84–12]	35 [82–12]	39 [86–16]	0.761
**Medications**				
**Aspirin**	40 (38%)	24 (38%)	16 (38%)	1.000
**Clopidogrel**	7 (7%)	4 (6%)	3 (7%)	1.000
**Warfarin**	3 (3%)	2 (3%)	1 (2%)	1.000
**Statin**	39 (37%)	25 (40%)	14 (33%)	0.543
**Beta blocker**	60 (57%)	36 (57%)	24 (57%)	1.000
**Calcium channel blocker**	41 (39%)	23 (37%)	18 (43%)	0.545
**ACE inhibitors**	5 (5%)	3 (5%)	2 (5%)	1.000
**ARB**	40 (38%)	23 (37%)	17 (40%)	0.688
**Spironolactone**	2 (2%)	1 (2%)	1 (2%)	1.000
**Furosemide**	57 (54%)	36 (57%)	21 (50%)	0.550
**Hydralazine**	22 (21%)	11 (17%)	11 (26%)	0.331
**Amiodarone**	3 (3%)	2 (3%)	1 (2%)	1.000
**Sevelamer**	8 (8%)	4 (6%)	4 (10%)	0.711
**Vitamin D**	8 (8%)	7 (11%)	1 (2%)	0.140
**Calcium carbonate**	26 (25%)	19 (30%)	7 (17%)	0.166
**Insulin**	38 (36%)	19 (30%)	19 (45%)	0.148
**Iron (enteral or parenteral)**	16 (15%)	9 (14%)	7 (16%)	0.784
**Systolic pressure (mmHg)**	140 [130–160]	140 [130–159]	148 [120–160]	0.598
**Diastolic pressure (mmHg)**	80 [70–90]	80 [70–90]	80 [70–90]	0.530
**Mean pressure (mmHg)**	100 [93–110]	98 [91–110]	103 [93–113]	0.500
**Respiratory rate (bpm)**	20 ± 4	20 ± 4	21 ± 4	0.179
**Oxygen saturation (%)**	93 ± 6	93 ± 6	94 ± 6.1	0.351
**F_i_O_2_ (%)**	28 ± 14.6	28 ± 15.9	27 ± 12.6	0.744
**Temperature (ºC)**	37 ± 1	37 ± 1	36 ± 1	0.374

Abbreviations – NID: non-insulin-dependent; ID: insulin-dependent; AMI: acute myocardial infarction; COPD: chronic obstructive pulmonary disease; AIDS: acquired immunodeficiency syndrome; CKD: chronic kidney disease; Fi02: fraction of inspired oxygen; BPM = breaths per minute.

**Table 2:** Alternative diagnosis for patients without COVID-19

Diagnosis	Number (%) N = 46
**Pulmonary congestion**	24 (52,2)
**Pneumonia**	14 (30.4)
**Dialysis catheter infection**	10 (21.7)
**Acute coronary syndrome**	3 (6.5)
**Septic arthritis**	1 (2.2)
**Empyema**	1 (2.2)
**Pleuroperitoneal fistula**	1 (2.2)
**COPD exacerbation**	1 (2.2)
**Bloodstream infection**	1 (2.2)
**Endocarditis**	1 (2.2)
**Mesenteric venous thrombosis**	1 (2.2)

Abbreviation – COPD: chronic obstructive pulmonary disease.

At ED admission, median systolic and diastolic blood pressure were 140 (IQR = 130-160) mmHg and 80 (IQR = 70–90) mmHg, respectively. Mean respiratory rate was 20 ± 4 breaths per minute and mean oxygen saturation was 93 ± 6%, with a fraction of inspired oxygen of 28 ± 14.6%. All vital signs were balanced in both groups (Table 1).

### Pulmonary Computed Tomography Findings

In general, Chest CT was done in the fifth day of symptoms, as shown in Table 3. The most common pulmonary lesion on CT was ground glass opacities (GGO). COVID-19-positive patients presented more frequently with the following pattern of GGO: peripheral distribution (42 (64%) vs 2 (5%),*p* < 0.001), bilateral/multifocal (42 (64%) vs 2 (5%),*p* < 0.001), and involvement of inferior/middle lung lobes (23 (35%) vs 4 (9%,*p* = 0.003). On the other hand, COVID-19-negative patients were more likely to present with smooth septal thickness (5 (8%) vs 11 (26%),*p* = 0.013) and pleural effusion (30 (45%) vs 32 (74%),*p* = 0.003). Unilateral GGO, 2 (3%) vs 2 (5%),*p* = 0.646, centrilobular nodules, 5 (8%) vs 4 (9%),*p* = 0.737, pericardial effusion, 9 (14%) vs 7 (16%),*p* = 0.784, and cardiomegaly, 30 (45%) vs 20 (47%),*p* = 1.000, were equally found in both groups. Tree-in-bud sign was rarely seen in this cohort.

**Table 3:** Computed tomography findings in the study population

Tomographic characteristic	All participants (109)	COVID-19 + (66)	COVID-19 – (43)	*p* value
Days between CT and initial symptoms	3; 4 [6–2]	4; 5 [7–2]	3; 4 [5–1]	0.226
Tomographic pattern				
Typical CT	40 (37%)	40 (61%)	0 (0%)	<0.001
Atypical CT	33 (30%)	9 (14%)	24 (56%)	<0.001
Indeterminate CT	19 (17%)	13 (20%)	6 (14%)	0.606
Negative CT	17 (16%)	4 (6%)	12 (28%)	0.003
GGO				
Peripheral	44 (40%)	42 (64%)	2 (5%)	<0.001
Bilateral/multifocal	54 (50%)	47 (71%)	7 (16%)	<0.001
Inferior/middle	27 (25%)	23 (35%)	4 (9%)	0.003
Perihilar	16 (15%)	9 (14%)	7 (16%)	0.784
Unilateral	4 (4%)	2 (3%)	2 (5%)	0.646
Smooth septal thickness	16 (15%)	5 (8%)	11 (26%)	0.013
Pleural effusion	62 (57%)	30 (45%)	32 (74%)	0.003
Centrilobular nodules	9 (8%)	5 (8%)	4 (9%)	0.737
Pericardial effusion	16 (15%)	9 (14%)	7 (16%)	0.784
Cardiomegaly	50 (46%)	30 (45%)	20 (47%)	1.000
Tree-in-bud sign	1 (1%)	0 (0%)	1 (2%)	0.394

Abbreviations – CT: computed tomography; GGO: ground glass opacity.

Regarding the classification of CT patterns, most COVID-19-positive patients had a typical pattern compared to COVID-19-negative patients (40 (61%) vs 0 (0%),*p* < 0.001). With RT-PCR result as reference, a typical image for COVID-19 had sensitivity of 60.61% (40/66) and specificity of 100% (40/40). PPV and NPV were 100% and 62.3%, respectively (Table 4). It was not possible to calculate positive likelihood ratio, once specificity was 100%. However, the negative likelihood ratio (NLR) was 0.39. Other statistical tests are described in Table 4. Atypical CT pattern was more frequent in COVID-19-negative patients (9 (14%) vs 24 (56%),*p* < 0.001), while the indeterminate pattern was similar in both groups (13 (20%) vs 6 (14%),*p* = 0.606) and the negative pattern was more common in COVID-19-negative patients (4 (6%) vs 12 (28%),*p* = 0.002) (Figure 1).

**Table 4:** Cross tabulation of the ct findings by the results of RT-PCR and performance for COVID-19 diagnosis in hospitalized ESKD patients on RRT

	RT-PCR +	RT-PCR –	Total
Typical CT	40	0	40
Non typical CT	26	43	82
Total	66	43	109
Statistic	**Value**	**95% CI**	
Sensitivity	60.61%	47.81–72.42%	
Specificity	100.00%	91.78–100.00%	
Positive Likelihood Ratio	NA	–	
Negative Likelihood Ratio	0.39	0.29-0.53	
Positive Predictive Value	100.00%	–	
Negative Predictive Value	62.32%	55.08–69.05%	
Accuracy	76.15%	67.03–83.79%	

Abbreviations – CI: confidence interval; CT: computed tomography; NA: not applicable.

**Figure 3.**
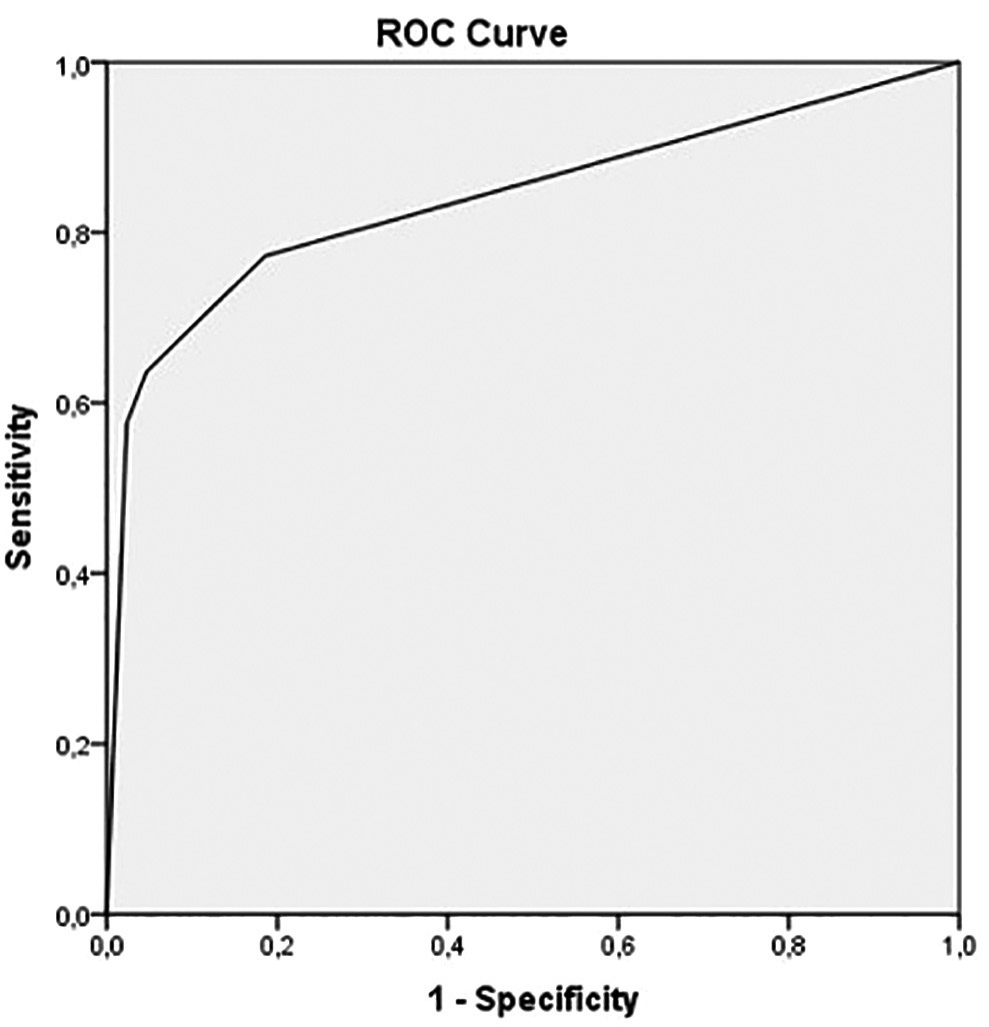
Area under the ROC curve for the predicted model.

Multivariate logistic regression showed that only peripheral GGO (OR 16.59, CI = 3.3–82.9,*p* = 0.001) and bilateral/multifocal GGO (OR 4.01, CI = 1.3–12.4,*p* = 0.016) were independently associated with COVID-19 diagnosis in ESKD patients on RRT. The area under the ROC curve for the diagnosis of COVID-19 was 0.84 (0.77-0.92) (Figure 3).

## Discussion

The current study shows that ESKD patients on RRT with suspicion of COVID-19 may have indistinguishable clinical presentation from other respiratory diagnoses, since baseline characteristics and vital signs were similar, except for current smoking (Table 1). This difference in current tobacco exposure is probably a spurious relationship given the small number of patients, and we believe it didn’t affect the results. Therefore, for the evaluation of these patients in ED, CT may have an important role in diagnosis, identify pulmonary complications and the extent of pulmonary involvement. To our knowledge, this is the first study that systematically evaluated CT patterns of ESKD patients on RRT with clinical suspicion of COVID-19, a commonly neglected population in clinical studies^20^. Abrishami et al. have described radiologic patterns in 43 ESKD patients, but only 5 were on dialysis^21^. ED physicians should pay special attention to these patients, since the higher prevalence of comorbidities may lead to clinical deterioration.

In this cohort, hospitalized ESKD patients on RRT with confirmed COVID-19 commonly presented with typical CT findings of SARS-CoV-2 infection. The most common CT finding was peripheral GGO. However, atypical CT imaging, such as pleural and pericardial effusion and cardiomegaly, appeared in similar proportions in COVID-19-positive and -negative patients. The sensitivity and specificity of a typical CT were respectively 60.6% and 100% in this population. The PPV was 100% and NPV was 62.3%. Multivariate logistic regression showed that only peripheral and bilateral/multifocal GGO were associated with a positive RT-PCR for SARS-CoV-2, confirming the classification according to guidelines^17^.

Although most of the confirmed COVID-19 patients had a typical CT finding, almost 40% had different pattern, which indicates that the use of CT as a screening tool is less effective in ESKD patients on RRT. In such scenario, chest CT should have a near perfect sensitivity so that a negative result excludes COVID-19^22^. In our cohort, we found a relatively low sensitivity of typical CT (60.6%) compared to the general population (97%)^23^. The low NPV and higher than 0.1 NLR reflect the inability of CT to rule-out COVID-19 in ESKD patients, even in the context of high suspicion and prevalence of the disease. The reason for that may be the higher frequency of atypical signs in this population, including cardiomegaly, pleural and pericardial effusion. These alterations are well known causes of fluid overload related to chronic dialysis management^24^. Therefore, these common findings of ESKD patients on RRT may be confounding factors when interpreting CT findings for COVID-19 diagnosis. In addition, these patients also appear to have several prior chronic and acute pulmonary alterations that may also cast doubt on the interpretation of chest CT^10-12^. Another important issue is that the treatment of lung changes in COVID-19 is completely different from pulmonary congestion related to volume overload, which is very common in patients with ESKD. Taken together, these factors pose a challenge to clinicians regarding when to isolate ESKD patients admitted with suspected COVID-19, based only on CT findings when RT-PCR is not readily available. To overcome or at least minimize this problem, these patients should be referred to an intermediate ward until RT-PCR or an appropriate point-of-care antigen test is available.

Pulmonary congestion was the main diagnosis in patients without positive RT-PCR for SARS-CoV-2. Thus, clinicians should keep in mind that CT findings suggestive of fluid overload that improve significantly after the dialysis session could make the diagnosis of COVID-19 less likely. Another concern is the possibility of bacterial infection. For this reason, clinicians should consider drawing cultures and starting antibiotics as soon as possible.

Our study has several limitations. The observational nature of the study may increase the risk of selection bias. However, our population is very similar to ESKD patients on RRT worldwide, considering the mean age, gender distribution and the fact that diabetes is the main cause of CKD. The applicability of our findings to patients with other underlying diseases, such as glomerulonephritis, should be done with caution. Information bias is also a concern. Data relating to patient history, clinical findings during hospitalization, and CT scan results may have been misclassified. However, we believe that this bias was minimized, at least for the CT findings, by using experienced external radiologists to review the exams and classify them according to current guidelines^17^. Moreover, since this study was focused on a subpopulation of COVID-19 patients with more pronounced symptoms, mild cases might have gone undiagnosed or were not recommended for imaging. In this sense, our findings may only reflect more severe COVID-19 cases in ESKD.

The relatively small number of patients may have reduced the external validity of our findings. However, ESKD patients are usually underrepresented in clinical trials, and larger studies were unlikely to be conducted because of the pandemic. We did not include patients without ESKD as a comparison group, although CT data of this group were well described^23^. The present results reflect the first wave of the COVID-19 pandemic and may not be applicable to less virulent strains such as Omicron. However, coronaviruses are well known to undergo recombination, leading to new genotypes and outbreaks, making CT knowledge essential^25^.

## Conclusion

chest CT is a valuable diagnostic test for COVID-19. In hospitalized patients with ESKD on RRT, however, an atypical pattern cannot adequately rule out the diagnosis of COVID-19. In that sense, in cases with atypical classifications, chest CT findings should not be used to guide clinical decisions until more sensitive tests are available.
