# Supplementary material for: COVID-19 Computed tomography patterns in renal replacement therapy patients
Source: J Bras Nefrol. 2024 Mar 11;46(3):e20230029. doi: 10.1590/2175-8239-JBN-2023-0029en (PMC11300031; doi:10.1590/2175-8239-JBN-2023-0029en)
Supplement: Translation

# Translation: Padrões de tomografia computadorizada da COVID-19 em pacientes em terapia renal substitutiva

**DOI:** 10.1590/2175-8239-JBN-2023-0029pt

**Authors:** Gabriel Assis Lopes do Carmo, Mariana Paiva Oliveira, Anna Luiza Lino Campos, Bráulio Roberto Gonçalves Marinho Couto, Lilian Pires de Freitas do Carmo, Tiago Lemos Cerqueira, Camila Alencar Monteiro de Souza, Yan Lopes Goll, Vitor Santos de Souza, Mariana Oliveira Guimarães Vieira, Pedro Alves Soares Vaz de Castro, Pedro Augusto Botelho Lemos, Ana Cristina Simões e Silva

**Affiliations:** 1 Hospital Evangélico de Belo Horizonte, Belo Horizonte, MG, Brasil.; 2 Universidade Federal de Minas Gerais, Faculdade de Medicina, Departamento de Clínica Médica, Belo Horizonte, MG, Brasil.; 3 Hospital Felicio Rocho, Belo Horizonte, MG, Brasil.; 4 Universidade Federal de Minas Gerais, Faculdade de Medicina, Belo Horizonte, MG, Brasil.; 5 Universidade Federal de Minas Gerais, Faculdade de Medicina, Laboratório Interdisciplinar de Investigação Médica, Unidade de Nefrologia Pediátrica, Belo Horizonte, MG, Brasil.

**Keywords:** Tomografia computadorizada, COVID-19, Diálise, Pronto-socorro, Doença renal em estágio terminal

## Abstract

**Introdução:**

Doenças pulmonares são comuns em pacientes com doença renal em estágio terminal (DRET), dificultando o diagnóstico diferencial com COVID-19. Este estudo descreve achados de tomografia computadorizada de tórax (TC) em pacientes com DRET em terapia renal substitutiva (TRS) hospitalizados com suspeita de COVID-19.

**Métodos:**

Indivíduos maiores de 18 anos com DRET, encaminhados ao pronto-socorro com suspeita de COVID-19 foram incluídos. Dados clínicos e epidemiológicos foram extraídos de registros eletrônicos de saúde. A TC foi classificada como típica, indeterminada, atípica, negativa. Comparamos achados tomográficos de pacientes com COVID-19 positivos e negativos.

**Resultados:**

Recrutamos 109 pacientes (62,3% COVID-19-positivos) entre março e dezembro de 2020, idade média de 60 ± 12,5 anos, 43% mulheres. A etiologia mais comum da DRET foi diabetes. Tempo médio em diálise foi 36 meses, intervalo interquartil = 12–84. A lesão pulmonar mais comum foi opacidades em vidro fosco. O padrão típico de TC foi mais comum em pacientes com COVID-19 (40 (61%) vs. 0 (0%) em pacientes sem COVID-19, *p* < 0,001). Sensibilidade 60,61% (40/66), especificidade 100% (40/40). Valores preditivos positivos e negativos foram 100% e 62,3%, respectivamente. Padrão atípico de TC foi mais frequente em pacientes COVID-19-negativos (9 (14%) vs. 24 (56%) em COVID-19-positivos, *p* < 0,001), enquanto padrão indeterminado foi semelhante em ambos os grupos (13 (20%) vs. 6 (14%), *p* = 0,606), e padrão negativo foi mais comum em pacientes COVID-19-negativos (4 (6%) vs. 12 (28%), *p* = 0,002).

**Conclusões:**

Em pacientes com DRET em TRS hospitalizados, um padrão atípico de TC de tórax não pode excluir adequadamente o diagnóstico de COVID-19.

## Introdução

A Doença do Coronavírus 2019 (COVID-19), causada pelo Coronavírus 2 da Síndrome Respiratória Aguda Grave (SARS-CoV-2), foi descrita pela primeira vez em Wuhan, China, em dezembro de 2019^1^. Até o momento, o vírus infectou mais de 800 milhões de pessoas em todo o mundo, causando mais de 6 milhões de óbitos^2^. Semelhante ao SARS-CoV e à Síndrome Respiratória do Oriente Médio (MERS), os sintomas são principalmente respiratórios, e as formas graves representam até 20% dos casos^3^. Idade avançada, obesidade, hipertensão, diabetes, doenças cardíacas, pulmonares e renais crônicas subjacentes são condições clínicas relacionadas a um pior prognóstico^4,5^.

Os pacientes com doença renal em estágio terminal (DRET) em terapia renal substitutiva (TRS) são particularmente preocupantes, uma vez que compartilham muitas dessas comorbidades e estão altamente expostos. Esses pacientes viajam frequentemente de e para instalações de hemodiálise e precisam se reunir diversas vezes por semana em um ambiente fechado^6^. Relatórios preliminares mostraram um aumento no risco de óbito para essa população^7–9^. Além disso, a apresentação clínica de pacientes com DRET com doenças respiratórias no pronto-socorro (PS) também é desafiadora, uma vez que eles podem apresentar diversas condições pulmonares subjacentes^10-12^. Tais doenças podem ter achados clínicos, laboratoriais e radiológicos semelhantes que geralmente ajudam no diagnóstico da COVID-19^13^. Portanto, é fundamental definir os achados radiológicos que permitem o diagnóstico precoce da COVID-19 em pacientes com DRET no PS, a fim de tratá-los e isolá-los de forma adequada. Nesse contexto, a tomografia computadorizada (TC) é de particular importância para essa avaliação. A TC pode mostrar um padrão pulmonar típico que levanta suspeita de infecção por SARS-CoV-2, mesmo quando o RT-PCR é negativo^13^. Além disso, as TCs sequenciais durante o curso da doença do paciente podem detectar complicações e prever o prognóstico^14,15^.

Os objetivos deste estudo foram descrever os achados de TC pulmonar em pacientes com DRET em TRS encaminhados ao PS com suspeita clínica de COVID-19, comparar características de imagem de casos positivos para COVID-19, confirmados por testes RT-PCR, com casos negativos para COVID-19 e verificar se esses resultados de TC em pacientes com DRET e COVID-19 apresentam boa sensibilidade e especificidade para diagnosticar a COVID-19 sem testes moleculares específicos. Essa análise é parte do “Estudo prospectivo da COVID-19 em pacientes dialíticos (VIDA)”, que atualmente está recrutando casos (número ReBEC RBR-63hzd3, disponível em http://www.ensaiosclinicos.gov.br/rg/RBR-63hzd3/).

## Métodos

### Desenho do Estudo

O estudo VIDA é uma coorte multicêntrica retrospectiva e prospectiva de pacientes com DRET em TRS com o objetivo de avaliar o impacto da COVID-19 nessa população. Indivíduos de ambos os sexos e maiores de 18 anos foram recrutados em 4 clínicas de diálise da Associação Evangélica Beneficente de Minas Gerais, em Belo Horizonte, Brasil, desde março de 2020.

### Critérios de Inclusão

O nefrologista assistente avaliou os pacientes incluídos na coorte VIDA com suspeita de COVID-19 durante a sessão de diálise para decidir se era necessário o encaminhamento ao pronto-socorro (PS). Nessa subanálise, selecionamos pacientes com sintomas respiratórios encaminhados ao PS entre março e dezembro de 2020. Durante esse período, as linhagens virais mais prevalentes no Brasil foram a B.1.1.28 e a B.1.1.33^16^. Não foi realizado nenhum cálculo de tamanho de amostra por se tratar de uma amostra de conveniência.

### Questões Éticas

O estudo foi realizado em conformidade com a Declaração de Helsinque. O conselho de revisão local aprovou nosso estudo e todos os pacientes assinaram um consentimento informado por escrito (número do Conselho de Revisão Institucional 31017120.9.3001.5149). O estudo está registrado sob o número ReBEC RBR-63hzd3, e o protocolo completo do estudo VIDA está disponível em http://www.ensaiosclinicos.gov.br/rg/RBR-63hzd3/.

### Protocolo de Estudo

As informações clínicas basais epidemiológicas e os sinais vitais foram extraídos dos registros eletrônicos de saúde. Com base nesses dados, calculamos o escore de Charlson. Selecionamos indivíduos que apresentaram TC pulmonar e swab nasofaríngeo para o teste RT-PCR SARS-Cov-2 para confirmar o diagnóstico de COVID-19. A TC pulmonar foi interpretada por dois radiologistas experientes e classificada como típica, indeterminada, atípica ou negativa para COVID-19 com base nas diretrizes atuais (Figura 1)^17^. A análise principal foi a comparação de achados de TC de pacientes com COVID-19 positivos e negativos.

**Figura 1.** Padrões de tomografia computadorizada de tórax na COVID-19. (A) COVID-19 típica: opacidade em vidro fosco periférica e bilateral, consolidação e linhas intralobulares visíveis (“pavimentação em mosaico”); (B) Indeterminada: opacidade em vidro fosco bilateral, especialmente nos lobos inferiores e derrame pleural; (C) Atípica: opacidade em vidro fosco central, espessamento do feixe broncovascular e espessamento do septo interlobular, sugerindo edema pulmonar intersticial; (D) Semelhante à imagem C mais derrame pleural e cardiomegalia, sugerindo edema pulmonar hidrostático; (E) Atípica: consolidação isolada com áreas de opacidades em vidro fosco; (F) Negativa: Sem características de TC que sugiram pneumonia.

### Análise Estatística

Os dados do estudo foram coletados e manejados usando ferramentas eletrônicas de captura de dados*REDCap* ^18,19^. As variáveis numéricas são apresentadas como média ± desvio padrão (DP) ou, no caso de distribuição não gaussiana, como valores medianos e intervalos. Os dados qualitativos são apresentados como porcentagem. Os grupos de estudo (COVID-19+*versus* COVID-19-) foram comparados em uma análise univariada por testes estatísticos de hipóteses bicaudal, considerando um nível de significância de 5%. Comparamos as variáveis qualitativas usando os testes exato de Fisher ou qui-quadrado. Para dados quantitativos, foram utilizados os testes de Wilcoxon ou Mann-Whitney. Foram calculados sensibilidade, especificidade, valor preditivo positivo (VPP) e valor preditivo negativo (VPN), bem como curvas ROC dos padrões de imagem de TC para diagnóstico confirmado de COVID-19 por RT-PCR. As variáveis com valores de*p* ≤ 0,25 na análise univariada foram incluídas em uma análise multivariada por regressão logística para identificar associações independentes com o diagnóstico de COVID-19. As associações foram relatadas como*odds ratio* (OR) com seus respectivos intervalos de confiança (IC) de 95%, bem como o teste de significância estatística. Não realizamos nenhuma análise estatística para dados perdidos. Para todas as análises estatísticas, utilizamos o software SPSS (versão 20).

## Resultados

### Características Basais dos Pacientes

De março a dezembro de 2020, 122 pacientes com DRET em TRS foram hospitalizados em nossa instituição com suspeita clínica de COVID-19. Um total de 76 indivíduos (62,3%) testou positivo para COVID-19, enquanto os 46 restantes apresentaram outro diagnóstico (grupo alternativo). Dez pacientes do grupo COVID-19 e 3 do grupo alternativo não realizaram TC de tórax e foram excluídos (Figura 2).

**Figura 2.** Fluxograma de registro.

As características clínicas e demográficas de toda a coorte do estudo estão resumidas na Tabela 1. A média de idade da coorte foi de 60 ± 12,5 anos, 43% eram mulheres, e a etiologia mais comum da DRET foi o diabetes, seguido por hipertensão. O tempo mediano em diálise foi de 36 meses (intervalo interquartil (IIQ) de 12 a 84), e o valor médio do escore prognóstico de Charlson foi de 5 ± 34,1 (33% de sobrevida estimada em 10 anos). As características clínicas foram semelhantes entre os pacientes positivos e negativos para a COVID-19, exceto pelo uso atual de tabaco (0%*vs.* 9%;*p* = 0,022, para COVID-19-positivo e COVID-19-negativo, respectivamente) (Tabela 1). O principal diagnóstico alternativo para pacientes sem COVID-19 foi congestão pulmonar, seguido por pneumonia e infecção da corrente sanguínea relacionada ao cateter de diálise. A maioria dos pacientes apresentou mais de um diagnóstico, como mostrado na Tabela 2.

**Tabela 1:** Características basais da população do estudo

Característica	Todos os participantes (109)	COVID-19 + (66)	COVID-19 – (43)	Valor de *p*
**Idade**	60 ± 12,5	60 ± 12,8	59 ± 12,3	0,794
**Sexo feminino**	47 (43%)	27 (41%)	20 (47%)	0,693
**Hipertensão**	103 (95%)	61 (92%)	42 (98%)	0,400
**Diabetes Mellitus NDI**	10 (9%)	7 (11%)	3 (7%)	0,737
**Diabetes Mellitus DI**	50 (46%)	29 (44%)	21 (49%)	0,695
**Uso atual de tabaco**	4 (4%)	0 (0%)	4 (9%)	0,022
**Uso prévio de tabaco**	12 (11%)	8 (12%)	4 (9%)	0,761
**Doença arterial coronariana**	14 (13%)	6 (9%)	8 (19%)	0,158
**Insuficiência cardíaca**	16 (15%)	9 (14%)	7 (16%)	0,784
**IAM prévio**	9 (8%)	5 (8%)	4 (9%)	0,737
**Doença cerebrovascular**	15 (14%)	8 (12%)	7 (16%)	0,578
**Doença arterial periférica**	4 (4%)	2 (3%)	2 (5%)	0,646
**DPOC**	8 (7%)	3 (5%)	5 (12%)	0,260
**Asma**	5 (5%)	1 (2%)	4 (9%)	0,078
**AIDS**	1 (<1%)			
**Escore de Charlson**				
**Numérico**	5 ± 1,8	5 ± 1,8	5 ± 1,7	0,968
**Percentual**	32 ± 33,7	32 ± 33,6	32 ± 34,3	0,950
**Escore de Charlson (%)**	33 ± 34,1			
**Etiologia da DRC**				
**Diabetes**	50 (41%)	32 (42%)	18 (39%)	0,145
**Hipertensão**	27 (22%)	15 (20%)	12 (26%)	
**Glomerulopatia**	15 (12%)	13 (17%)	2 (4%)	
**Outros**	30 (25%)	16 (21%)	14 (30%)	
**Tempo em diálise (meses)**	36 [84–12]	35 [82–12]	39 [86–16]	0,761
**Medicamentos**				
**Aspirina**	40 (38%)	24 (38%)	16 (38%)	1,000
**Clopidogrel**	7 (7%)	4 (6%)	3 (7%)	1,000
**Varfarina**	3 (3%)	2 (3%)	1 (2%)	1,000
**Estatina**	39 (37%)	25 (40%)	14 (33%)	0,543
**Betabloqueador**	60 (57%)	36 (57%)	24 (57%)	1,000
**Bloqueador de canais de cálcio**	41 (39%)	23 (37%)	18 (43%)	0,545
**Inibidores da ECA**	5 (5%)	3 (5%)	2 (5%)	1,000
**BRA**	40 (38%)	23 (37%)	17 (40%)	0,688
**Espironolactona**	2 (2%)	1 (2%)	1 (2%)	1,000
**Furosemida**	57 (54%)	36 (57%)	21 (50%)	0,550
**Hidralazina**	22 (21%)	11 (17%)	11 (26%)	0,331
**Amiodarona**	3 (3%)	2 (3%)	1 (2%)	1,000
**Sevelâmer**	8 (8%)	4 (6%)	4 (10%)	0,711
**Vitamina D**	8 (8%)	7 (11%)	1 (2%)	0,140
**Carbonato de cálcio**	26 (25%)	19 (30%)	7 (17%)	0,166
**Insulina**	38 (36%)	19 (30%)	19 (45%)	0,148
**Ferro (enteral ou parenteral)**	16 (15%)	9 (14%)	7 (16%)	0,784
**Pressão sistólica (mmHg)**	140 [130–160]	140 [130–159]	148 [120–160]	0,598
**Pressão diastólica (mmHg)**	80 [70–90]	80 [70–90]	80 [70–90]	0,530
**Pressão média (mmHg)**	100 [93–110]	98 [91–110]	103 [93–113]	0,500
**Frequência respiratória (bpm)**	20 ± 4	20 ± 4	21 ± 4	0,179
**Saturação de oxigênio (%)**	93 ± 6	93 ± 6	94 ± 6,1	0,351
**F_i_O_2_ (%)**	28 ± 14,6	28 ± 15,9	27 ± 12,6	0,744
**Temperatura (ºC)**	37 ± 1	37 ± 1	36 ± 1	0,374

Abreviações – NDI: não dependente de insulina; DI: dependente de insulina; IAM: infarto agudo do miocárdio; DPOC: doença pulmonar obstrutiva crônica; AIDS: síndrome da imunodeficiência adquirida; DRC: doença renal crônica; Fi02: fração inspirada de oxigênio; RPM = respirações por minuto.

**Tabela 2:** Diagnóstico alternativo para pacientes sem COVID-19

Diagnóstico	Número (%) N = 46
**Congestão pulmonar**	24 (52,2)
**Pneumonia**	14 (30,4)
**Infecção do cateter de diálise**	10 (21,7)
**Síndrome coronariana aguda**	3 (6,5)
**Artrite séptica**	1 (2,2)
**Empiema**	1 (2,2)
**Fístula pleuroperitoneal**	1 (2,2)
**Exacerbação da DPOC**	1 (2,2)
**Infecção da corrente sanguínea**	1 (2,2)
**Endocardite**	1 (2,2)
**Trombose venosa mesentérica**	1 (2,2)

Abreviação – DPOC: doença pulmonar obstrutiva crônica.

Na admissão ao PS, as medianas da pressão arterial sistólica e diastólica foram de 140 (IIQ = 130–160) mmHg e 80 (IIQ = 70–90) mmHg, respectivamente. A frequência respiratória média foi de 20 ± 4 respirações por minuto e a saturação média de oxigênio foi de 93 ± 6%, com uma fração inspirada de oxigênio de 28 ± 14,6%. Todos os sinais vitais estavam equilibrados em ambos os grupos (Tabela 1).

### Achados da Tomografia Computadorizada Pulmonar

Em geral, a TC de tórax foi realizada no quinto dia de sintomas, como mostra a Tabela 3. A lesão pulmonar mais comum na TC foram as opacidades em vidro fosco (OVF). Os pacientes positivos para COVID-19 apresentaram com maior frequência o seguinte padrão de OVF: distribuição periférica (42 (64%)*vs.* 2 (5%);*p* < 0,001), bilateral/multifocal (42 (64%)*vs.* 2 (5%);*p* < 0,001) e envolvimento dos lobos pulmonares inferiores/médios (23 (35%)*vs.* 4 (9%;*p* = 0,003). Por outro lado, os pacientes negativos para COVID-19 tiveram maior probabilidade de apresentar espessura septal lisa (5 (8%)*vs.* 11 (26%);*p* = 0,013) e derrame pleural (30 (45%)*vs.* 32 (74%);*p* = 0,003). OVF unilateral, 2 (3%)*vs.* 2 (5%);*p* = 0,646, nódulos centrolobulares, 5 (8%)*vs.* 4 (9%);*p* = 0,737, derrame pericárdico, 9 (14%)*vs.* 7 (16%);*p* = 0,784, e cardiomegalia, 30 (45%)*vs.* 20 (47%);*p* = 1,000, foram igualmente observados em ambos os grupos. O padrão de árvore em brotamento foi raramente observado nessa coorte.

**Tabela 3:** Achados de tomografia computadorizada na população do estudo

Característica tomográfica	Todos os participantes (109)	COVID-19 + (66)	COVID-19 – (43)	Valor de *p*
Dias entre a TC e os sintomas iniciais	3; 4 [6–2]	4; 5 [7–2]	3; 4 [5–1]	0,226
Padrão tomográfico				
TC típica	40 (37%)	40 (61%)	0 (0%)	<0,001
TC atípica	33 (30%)	9 (14%)	24 (56%)	<0,001
TC indeterminada	19 (17%)	13 (20%)	6 (14%)	0,606
TC negativa	17 (16%)	4 (6%)	12 (28%)	0,003
OVF				
Periférica	44 (40%)	42 (64%)	2 (5%)	<0,001
Bilateral/multifocal	54 (50%)	47 (71%)	7 (16%)	<0,001
Inferior/média	27 (25%)	23 (35%)	4 (9%)	0,003
Peri hilar	16 (15%)	9 (14%)	7 (16%)	0,784
Unilateral	4 (4%)	2 (3%)	2 (5%)	0,646
Espessura septal lisa	16 (15%)	5 (8%)	11 (26%)	0,013
Derrame pleural	62 (57%)	30 (45%)	32 (74%)	0,003
Nódulos centrilobulares	9 (8%)	5 (8%)	4 (9%)	0,737
Derrame pericárdico	16 (15%)	9 (14%)	7 (16%)	0,784
Cardiomegalia	50 (46%)	30 (45%)	20 (47%)	1,000
Padrão de árvore em brotamento	1 (1%)	0 (0%)	1 (2%)	0,394

Abreviações – TC: tomografia computadorizada; OVF: opacidade em vidro fosco.

Em relação à classificação dos padrões de TC, a maioria dos pacientes positivos para COVID-19 apresentou um padrão típico em comparação com os pacientes negativos para COVID-19 (40 (61%)*vs.* 0 (0%);*p* < 0,001). Com o resultado do RT-PCR como referência, uma imagem típica para a COVID-19 apresentou sensibilidade de 60,61% (40/66) e especificidade de 100% (40/40). O VPP e o VPN foram de 100% e 62,3%, respectivamente (Tabela 4). Não foi possível calcular a razão de verossimilhança positiva, uma vez que a especificidade foi de 100%. Entretanto, a razão de verossimilhança negativa (RVN) foi de 0,39. Outros testes estatísticos estão descritos na Tabela 4. O padrão atípico de TC foi mais frequente em pacientes negativos para COVID-19 (9 (14%)*vs.* 24 (56%);*p* < 0,001), enquanto o padrão indeterminado foi semelhante em ambos os grupos (13 (20%)*vs.* 6 (14%);*p* = 0,606) e o padrão negativo foi mais comum em pacientes negativos para COVID-19 (4 (6%)*vs.* 12 (28%);*p* = 0,002) (Figura 1).

**Tabela 4:** Tabulação cruzada dos achados de TC pelos resultados de RT-PCR e desempenho para o diagnóstico de COVID-19 em pacientes com DRET em TRS hospitalizados

	RT-PCR +	RT-PCR –	Total
TC típica	40	0	40
TC não típica	26	43	82
Total	66	43	109
Estatísticas	**Valor**	**IC 95%**	
Sensibilidade	60,61%	47,81–72,42%	
Especificidade	100,00%	91,78–100,00%	
Razão de Verossimilhança Positiva	NA	–	
Razão de Verossimilhança Negativa	0,39	0,29–0,53	
Valor Preditivo Positivo	100,00%	–	
Valor Preditivo Negativo	62,32%	55,08–69,05%	
Precisão	76,15%	67,03–83,79%	

Abreviações – IC: intervalo de confiança; TC: tomografia computadorizada; NA: não aplicável.

A regressão logística multivariada mostrou que apenas a OVF periférica (OR 16,59; IC = 3,3–82,9;*p* = 0,001) e a OVF bilateral/multifocal (OR 4,01; IC = 1,3-12,4;*p* = 0,016) foram independentemente associadas ao diagnóstico de COVID-19 em pacientes com DRET em TRS. A área sob a curva ROC para o diagnóstico da COVID-19 foi de 0,84 (0,77–0,92) (Figura 3).

**Figura 3.** Área sob a curva ROC para o modelo previsto.

## Discussão

O presente estudo mostra que pacientes com DRET em TRS com suspeita de COVID-19 podem ter uma apresentação clínica indistinguível de outros diagnósticos respiratórios, uma vez que as características basais e os sinais vitais foram semelhantes, exceto pelo tabagismo atual (Tabela 1). Essa diferença na exposição atual ao tabaco é provavelmente uma relação espúria, dado o pequeno número de pacientes, e acreditamos que não tenha afetado os resultados. Portanto, para a avaliação desses pacientes no PS, a TC pode ter um papel importante no diagnóstico, na identificação de complicações pulmonares e na extensão do envolvimento pulmonar. Até onde sabemos, este é o primeiro estudo que avaliou sistematicamente os padrões de TC de pacientes com DRET em TRS com suspeita clínica de COVID-19, uma população comumente negligenciada em estudos clínicos^20^. Abrishami et al. descreveram padrões radiológicos em 43 pacientes com DRET, mas apenas 5 estavam em diálise^21^. Os médicos do PS devem prestar atenção especial a esses pacientes, pois a maior prevalência de comorbidades pode levar à deterioração clínica.

Nessa coorte, os pacientes com DRET em TRS hospitalizados com COVID-19 confirmada geralmente apresentaram achados de TC típicos de infecção por SARS-CoV-2. O achado de TC mais comum foi a OVF periférica. No entanto, imagens de TC atípicas, como derrame pleural e pericárdico e cardiomegalia, apareceram em proporções semelhantes em pacientes positivos e negativos para a COVID-19. A sensibilidade e a especificidade de uma TC típica foram de 60,6% e 100%, respectivamente, nessa população. O VPP foi de 100% e o VPN foi de 62,3%. A regressão logística multivariada mostrou que somente a OVF periférica e bilateral/multifocal foi associada a um RT-PCR positivo para SARS-CoV-2, confirmando a classificação de acordo com as diretrizes^17^.

Embora a maioria dos pacientes com COVID-19 confirmada apresentasse um achado típico de TC, quase 40% apresentaram um padrão diferente, o que indica que o uso da TC como ferramenta de triagem é menos eficaz em pacientes com DRET em TRS. Nesse cenário, a TC de tórax deve ter uma sensibilidade quase perfeita, para que um resultado negativo exclua a COVID-19^22^. Em nossa coorte, encontramos uma sensibilidade relativamente baixa de TC típica (60,6%) em comparação com a população em geral (97%)^23^. O baixo VPN e uma RVN superior a 0,1 refletem a incapacidade da TC de descartar a COVID-19 em pacientes com DRET, mesmo no contexto de alta suspeita e prevalência da doença. A razão para isso pode ser a maior frequência de sinais atípicos nessa população, incluindo cardiomegalia, derrame pleural e pericárdico. Essas alterações são causas bem conhecidas de sobrecarga hídrica relacionada ao manejo da diálise crônica^24^. Portanto, esses achados comuns de pacientes com DRET em TRS podem ser fatores de confusão ao interpretar os achados da TC para o diagnóstico da COVID-19. Além disso, esses pacientes também parecem apresentar diversas alterações pulmonares crônicas e agudas prévias que também podem colocar em dúvida a interpretação da TC de tórax^10-12^. Outra questão importante é que o tratamento das alterações pulmonares na COVID-19 é completamente diferente da congestão pulmonar relacionada à sobrecarga de volume, muito comum em pacientes com DRET. Em conjunto, esses fatores representam um desafio para os médicos quanto ao momento de isolar os pacientes com DRET admitidos com suspeita de COVID-19, com base apenas nos achados de TC quando o RT-PCR não estiver prontamente disponível. Para superar ou pelo menos minimizar esse problema, esses pacientes devem ser encaminhados para uma enfermaria intermediária até que o RT-PCR ou um teste de antígeno apropriado no local de atendimento esteja disponível.

A congestão pulmonar foi o principal diagnóstico em pacientes sem RT-PCR positivo para SARS-CoV-2. Dessa forma, os médicos devem ter em mente que os achados de TC sugestivos de sobrecarga hídrica que melhoram significativamente após a sessão de diálise podem tornar o diagnóstico de COVID-19 menos provável. Outra preocupação é a possibilidade de infecção bacteriana. Por essa razão, os médicos devem considerar a coleta de culturas e o início do uso de antibióticos o mais rápido possível.

Nosso estudo apresenta diversas limitações. A natureza observacional do estudo pode aumentar o risco de viés de seleção. No entanto, nossa população é muito semelhante à de pacientes com DRET em TRS em todo o mundo, considerando a média de idade, a distribuição por sexo e o fato de o diabetes ser a principal causa de DRC. A aplicabilidade de nossos achados a pacientes com outras doenças subjacentes, como a glomerulonefrite, deve ser feita com cautela. O viés de informação também é uma preocupação. Os dados relacionados ao histórico do paciente, aos achados clínicos durante a hospitalização e aos resultados da TC podem ter sido classificados incorretamente. No entanto, acreditamos que esse viés foi minimizado, ao menos no que diz respeito aos achados da TC, com o uso de radiologistas externos experientes para revisar os exames e classificá-los de acordo com as diretrizes atuais^17^. Além disso, como este estudo se concentrou em uma subpopulação de pacientes com COVID-19 com sintomas mais pronunciados, os casos leves podem não ter sido diagnosticados ou não recomendados para exames de imagem. Nesse sentido, nossos achados podem refletir somente casos mais graves de COVID-19 na DRET.

O número relativamente pequeno de pacientes pode ter reduzido a validade externa de nossos achados. No entanto, pacientes com DRET geralmente são sub-representados em estudos clínicos, e era improvável que estudos maiores fossem realizados devido à pandemia. Não incluímos pacientes sem DRET como um grupo de comparação, embora os dados de TC desse grupo tenham sido bem descritos^23^. Os presentes resultados refletem a primeira onda da pandemia de COVID-19 e podem não ser aplicáveis a cepas menos virulentas, como a Omicron. No entanto, sabe-se que os coronavírus sofrem recombinação, o que leva a novos genótipos e surtos, tornando o conhecimento da TC essencial^25^.

## Conclusão

A TC de tórax é um teste diagnóstico valioso para a COVID-19. Em pacientes com DRET hospitalizados em TRS, no entanto, um padrão atípico não pode descartar adequadamente o diagnóstico de COVID-19. Nesse sentido, em casos com classificações atípicas, os achados de TC de tórax não devem ser utilizados para orientar decisões clínicas até que testes mais sensíveis estejam disponíveis.
